# Effect of Induced Oxidative Stress and Herbal Extracts on Acid Phosphatase Activity in Lysosomal and Microsomal Fractions of Midgut Tissue of the Silkworm, *Bombyx mori*

**DOI:** 10.1673/031.010.11301

**Published:** 2010-07-16

**Authors:** Y. B. Gaikwad, S. M. Gaikwad, G. P. Bhawane

**Affiliations:** ^1^ Department of Zoology, Shivaji University, Kolhapur, Maharashtra State, India; ^2^ Department of Zoology, Bhogawati College, Kurukali, Karveer, Kolhapur, Maharashtra State, India

**Keywords:** D-galactose, *Bacopa monniera* and *Lactuca sativa*

## Abstract

Lysosomal and microsomal acid phosphatase activity was estimated in midgut tissue of silkworm larvae, *Bombyx mori* L. (Lepidoptera: Bombycidae), after induced oxidative stress by D-galactose. The larvae were simultaneously were treated with ethanolic extracts of *Bacopa monniera* and *Lactuca sativa* to study their antioxidant properties. Lipid peroxidation and fluorescence was measured to analyze extent of oxidative stress. The ethanolic extract of *Lactuca sativa* was found to be more effective in protecting membranes against oxidative stress than *Bacopa monniera*.

## Introduction

Oxidative stress is caused by free radicals such as reactive oxygen species (ROS), which includes superoxide (O_2_^.-^), peroxyl, alkoxyl, hydroxyl and nitric oxide. ROS are characterized by presence of an unpaired electron in their outer orbit. In addition to these ROS radicals in living organisms, there are other ROS non-radicals such as the singlet oxygen (^1^O_2_), hydrogen peroxide and hypochlorous acid (Pietta, 2000). Small quantities of ROS are formed spontaneously under normal conditions as byproducts of redox processes such as oxidative phosphorylation in the mitochondria and βoxidation of fatty acids. However, the production of ROS is increased when the organism is subjected to irradiation, chemicals or infection (Knapowski *et al*. 2002). Overproduction of ROS damages cellular lipids, nucleic acids, proteins and leads to lipid peroxidation, genome instability or gene mutation; protein carbonyl formation and enzymatic inactivity resulting in degenerative processes leading to aging (Martin *et al*. 1996; Berlett and Standtman 1997; Finkel and Halbroook 2000). To defend against the ROS formed, animal cells use three enzymes, superoxide dismutase, catalase and glutathione peroxidase. Superoxide dismutase converts superoxide anion to oxygen and hydrogen peroxide. In biological tissues superoxide can also be converted nonenzymically into the nonradical species hydrogen peroxide and singlet oxygen (Steinbeck *et al*. 1993). Catalase reduces hydrogen peroxide to water and oxygen (Fridovich 1978). Glutathione peroxidase neutralizes hydrogen peroxide by taking hydrogens from two glutathione molecules resulting in two H2O and one molecule of an oxidized form of glutathione. Besides these cellular antioxidant enzymes there are non-enzyme antioxidants such as α-tocopherol, βcarotin, lycopene and ascorbic acid.

Evidence has accumulated showing that plant polyphenols are an important class of defense antioxidants. These compounds are widespread virtually in all plant foods, often at high levels, and include phenols, phenolic acids, flavonoids, tannins and lignans. Acid phosphatase (ACP) is a lysosomal enzyme that catalyzes the hydrolysis of variety of phosphate monoesters and phosphoprotein in an acidic medium. Phosphatases are capable of transphosphorylation in addition to hydrolysis. Phosphatases thus play an important role in the metabolism of carbohydrates, phospholipids and nucleotides (Hollander 1971; Van Rees 1888; Crossley 1964, 1965; Givindwar and Bhawane 1989, Bhawane and Bhanot 1989, 1990, 1994, Bhanot and Bhawane 1991). Acid phosphatase is found mainly in cytosol of midgut tissue cells of Diptera (Ferreira and Terra 1980) and Lepidoptera (Santos and Terra 1984). ACP activities in the blood and midgut tissues are strongly related to silk protein synthesis, digestion, and absorption of phosphorylating substances in silkworm larvae (Wu 1993). Malondialdehyde and 4-hydroxynonenal are the products of lipid peroxidation. Malondialdehyde and lipofuscin granules are the indicators of oxidative stress and increase with age (Nakano *et al*. 1995; Sitte *et al*. 2001).

In present investigation the silkworm *Bombyx mori* is used to study the effects of D-galactose-induced oxidative stress and the antioxidant properties of ethanolic extracts of *Bacopa monniera* and *Lactuca sativa*. Alcoholic extracts of *L. sativa* and *B. monniera* contain polyphenols and flavonoids.

It is well known that polyphenols and flavonoids have antioxidant properties. Previous studies suggest that antioxidant polyphenol compounds found in fruits and vegetables may reverse aging (Joseph *et al*. 2005). *L. sativa* contains quercetin a flavonol-type flavonoid ubiquitously present in fruits and vegetables (Bilyk and Supers 1985; Zhang, 2005). Quercetin has the antioxidant property of scavenging free radicals such as superoxide radicals, peroxyl and hydroxyl radicals generated in various cellular processes (Dok-Go *et al*. 2003; Ioku *et al*. 1995; Kefalas *et al*. 2003). *B. monniera* contains mixture of triterpenoid saponins and bacosides (Chatterjee *et al*. 1963). Previous studies on ethanolic extracts of *L. sativa* have shown neuroprotective effects by strengthening the antioxidant system of nervous cells in mice (Deshmukh *et al*. 2007). An ethanolic extract of *B. monniera* has shown to reduce lipid peroxidation and promotes antioxidant status (Rohini *et al*. 2004). In insects, the midgut is a dynamic tissue as it is the only section of the alimentary canal where the process of digestion and absorption of digested food takes place. Therefore this is the active organ in insect body organization selected in this study.

## Material and Methods

### Preparation of extracts

Fresh leaves of *Bacopa monniera* and *Lactuca sativa* were collected and washed with distilled water and shade dried. After complete drying, leaves were powdered and kept in alcohol for 72 hours for extraction. The alcohol was allowed to evaporate and the resulting paste was collected and stored for further use.

**Table 1.**
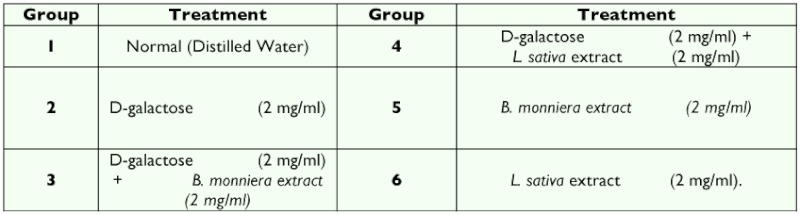
Groups and treatments.

### Silkworm rearing and administration of dose

The silkworm *B. mori* larvae of race PM were reared according to standard methods (Krishanaswami *et.al* 1978). Treatment was given feeding the fifth instar larvae for the first three days by dipping and air-drying mulberry leaves of equal weight in various solutions of doses prepared by dissolving Dgalactose and ethanolic herbal extracts (Table 1). Control mulberry leaves were dipped in distilled water. The larvae were treated for first three days of fifth instar. On the fourth day, midgut tissue was dissected from larvae.

### Sub-cellular fractionation

The midgut tissue was homogenized in 0.25 M sucrose containing 1mM EDTA. The homogenates were subjected to sub-cellular fractionation at 4° C. The homogenate was first centrifuged at 1,000 g to remove unbroken cells and nuclei. The pellet was discarded and the supernatant was again centrifuged at 10,000 g to separate lysosomes and mitochondria. The supernatant was used as microsomal enzyme source. The pellet was resuspended in 0.25M sucrose containing 1mM EDTA that served as lysosomal enzyme source.

### Measurement of lipid peroxidation and fluorescence

Lipid peroxidation was estimated using TCA-TBA-HCl reagent. The reaction mixture contained 1 ml homogenate prepared in 0.8% NaCl and 2 ml of TCA-TBA-HCl reagent. The tubes were kept in boiling water bath for 10 min. Tubes were removed and cooled and centrifuged, absorbance was measured at 532 nm using a spectrophotometer. Malondialdehyde was estimated using the extinction coefficient 1.56 × 10^5^/M MDA/cm^2^.

Fluorescence was measured using the Dillard and Tappel method (1971). One ml of homogenate was extracted at room temperature with 6 ml of chloroform and methanol mixture (2:1) the extracts were centrifuged for two min. at 1500 g. After mixing briefly with 6 ml of water two layers were formed. Four ml of the upper chloroform layer was removed to a test tube and 0.4 ml methanol was added. The fluorescence was determined using a photofluorometer calibrated with quinine sulfate at 1mg/ml.

**Figure 1.**
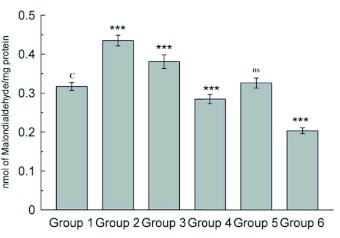
Lipid peroxidation in midgut tissue. Data are means ± SD (n = 6). µ*** shows highly significant change (p < 0.001), ** Shows significant change (p<0.05), **ns** shows non-significant change over the control group. High quality figures are available online.

### Estimation of ACP

The acid phosphatase activity was estimated using the Linhardt and Walter method. The assay mixture contained 0.2 ml of enzyme source and 0.8 ml of citrate buffer (pH 4.0) containing 5.5 × 10^-3^ M p-nitrophenyl phosphate as substrate. The assay tubes were incubated for 30 min. at 40° C. The reaction was stopped by adding 4 ml of 0.1 N NaOH. The absorbance was measured at 405 nm. Zero absorbance was adjusted by control devoid of enzyme source. The protein content in enzyme sources was estimated by the method of Lowry *et al*. (1951).

## Results and Discussion

**Figure 2.**
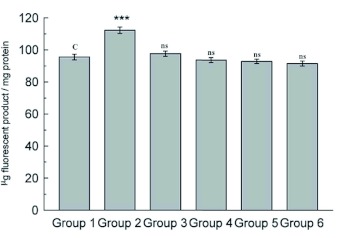
Estimation of fluorescent product in midgut tissue. Data are means ± SD (n = 6). *** shows highly significant change (p < 0.001), ** Shows significant change (p< 0.05), **ns** shows non-significant change over the control group. High quality figures are available online.

**Figure 3.**
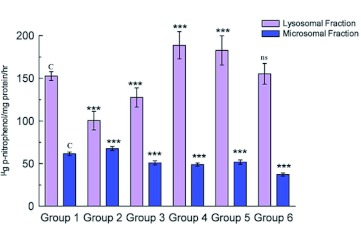
Estimation of ACP activity in lysosomal and microsomal fractions in midgut tissue. Data are means ± SD (n = 6). *** shows highly significant change (p < 0.001), ** Shows significant change (p< 0.05), **ns** shows non-significant change over the control group. High quality figures are available online.

D-galactose is a reducing sugar that accelerates the process of aging by increasing oxidative stress as was observed in *Drosophila melanogaster* and *Musca domestica* (Xu *et al*. 2004). With age, lysosomes of postmitotic cells increasingly become full with aggregates of oxidized, glycated and crosslinked proteins that are resistant to enzymatic degradation, which is called lipofuscin. Accumulation of lipofuscin is a major manifestation of aging in insects, the concentration of fluorescent age pigment increases with age in tissues (Sohal 1981). The results show a significant increase (p < 0.001) in midgut lipid peroxidation and fluorescent products in D-galactose treated larvae (Figures 1 and 2), indicating the increased oxidative stress due to D-galactose. Lysosomal acid phosphatase activity in midgut tissue of D- galactose treated larvae was decreased significantly, but the microsomal ACP activity was increased significantly (p < 0.001) as compared to the control group (Figure 3).

Cellular organelles are surrounded with lipid membranes that gets damaged due to lipid peroxidation which is result of oxidative stress. Damaged membrane looses its fluidity and integrity. The biological membranes can be disrupted by degrading phospholipids that are the primary molecular component of biological membranes (Tappel 1980; Donato and Sohal 1981). Lysosomal acid phosphatase is transported as a transmembrane protein to dense lysosomes, the pathway of lysosomal ACP to lysosomes include the passage through the plasma membrane (Martin *et al*. 1989).

Injured lysosomes release hydrolytic enzymes in cytoplasm leading to auto degradation of cellular proteins, damage to endoplasmic reticulum interferes with protein synthesis and intracellular transport of vital compounds (Zs-Nagy 1979 and 2002). Lipid peroxidation in membrane lipids plays an important role in cell physiology and pathology, there are number of membrane bound enzymes and their activity is altered by lipid peroxidation. Lipid peroxidation and fluorescent products were lower in midgut of larvae treated with herbal extract than the D-galactose treated group showing their antioxidant properties. There was no significant difference in fluorescent products as compared with control group. The ethanolic extract of *Bacopa monniera* was found to be less effective in protecting lipids against lipid peroxidation than the *Lactuca sativa* extract. Simultaneous treatments with extracts of *B. monniera* or *L. sativa* with D-galactose resulted in an increase in the lysosomal ACP activity (Figure 3) than D-galactose treated group, where as the microsomal ACP activity was significantly less.

The results showed that the ethanolic extract of *L. sativa* was more effective in protecting the lysosomal membrane integrity than *B. monniera*. Overall lysosomal ACP activity was found to be higher when there is a low level of oxidative stress. At higher levels of oxidative stress the lysosomal membrane was damaged that resulted in an increase in microsomal ACP activity.

## Abbreviations

**ACP**: acid phosphatase;
ROS: reactive oxygen species
